# The evaluation of Tenecteplase for the treatment of ischemic stroke (real-world data)

**DOI:** 10.3389/fneur.2026.1869006

**Published:** 2026-07-07

**Authors:** William Braun, Allan Weiss, Harold Colbassani, Ajay Arora, Paul Lewis, Eric Lopez del Valle, Marissa Lepain, Christi Newman, Keith Chastain

**Affiliations:** BayCare Health System, Clearwater, FL, United States

**Keywords:** ischemic stroke, large vessel occlusion, modified Rankin scale, Tenecteplase, thrombectomy

## Abstract

**Importance:**

The study reinforces the use of Tenecteplase (TNK) dosed at 0.25 mg/kg in real-world community settings as an effective and safe alternative to alteplase standard dosing.

**Objective:**

The aim of this study is to evaluate the effectiveness and safety of TNK compared to alteplase in a community healthcare setting for treating ischemic stroke, including patients with large vessel occlusion (LVO) who were candidates for mechanical thrombectomy, as well as those with non-large-vessel ischemic stroke.

**Design, setting, and participants:**

This was a multicenter, retrospective cohort study across 11 primary and comprehensive hospitals within the BayCare Health System in West Central Florida (December 2019–April 2024), comparing TNK 0.25 mg/kg (max 25 mg) with standard-dose alteplase among adults with acute ischemic stroke presenting within 4.5 h of last-known-well. A total of 476 patients were evaluated retrospectively, with 270 patients in the Alteplase arm and 206 patients in the TNK arm.

**Main outcome and measures:**

The primary outcome (LVO subgroup) was substantial early reperfusion prior to thrombectomy, defined as restoration of blood flow to >50% of the ischemic territory (mTICI 2b/2c/3) by CTA/MRA or absence of a retrievable thrombus at the initial angiographic assessment. A LVO was determined at the initial CT/CTA prior to the thrombectomy. Secondary outcomes included door-to-needle time (DTN), NIHSS at 24 h, discharge NIHSS, discharge disposition, 90-day mRS, and safety (symptomatic/asymptomatic ICH by system definitions and angioedema). Safety outcomes for ICH were assessed using the SITS-MOST criteria for symptomatic ICH including subarachnoid hemorrhage within 24 h of treatment combined with an increase from baseline in NIHSS score of at least 4 points.

**Results:**

Of the 476 AIS patients (TNK *n* = 270; alteplase *n* = 206), 226 had LVO (TNK *n* = 115; alteplase *n* = 111). Early reperfusion occurred in 14.8% treated with TNK vs. 4.5% with alteplase (risk difference 10.3% [95% CI, 2.7–17.8], *p* = 0.008). Adjusted odds of early reperfusion were higher with TNK (OR 3.53 [95% CI, 1.20–10.40], *p* = 0.022). In all AIS patients, median DTN time was shorter with TNK (34 vs. 45 min; difference −11 [95% CI, −14.4 to −7.6], *p* < 0.001). Good 90-day functional outcome (mRS 0–2) was more common with TNK among LVO patients (47.3% vs. 29.3%, *p* = 0.031) and among all AIS patients (61.8% vs. 50.0%, difference 11.8% [95% CI, −0.3 to 23.4], *p* = 0.044). Symptomatic ICH and angioedema were similar; across all patients combined, ICH (symptomatic and asymptomatic) was less frequent with TNK (8.5% vs. 15.0%, *p* = 0.030).

**Conclusion and relevance:**

In a community-hospital system, TNK 0.25 mg/kg IV was associated with higher pre-thrombectomy reperfusion in LVO, shorter door-to-needle times, comparable safety, and improved functional outcomes versus alteplase in key analyses. These findings align with recent randomized evidence and support TNK 0.25 mg/kg as an effective alternative to alteplase for AIS.

## Introduction

For more than 25 years since its Food and Drug Administration (FDA) approval in 1996, alteplase 0.9 mg/kg (maximum dose 90 mg with 10% given as a bolus) has been the standard of care for thrombolysis in the setting of Acute Ischemic Stroke (AIS). Tenecteplase (TNK) was engineered by modifying the Alteplase structure using point mutation of the Alteplase 527 amino acid glycoprotein structure at three sites: threonine 103, asparagine 117, and kringle 1 domain. These modifications resulted in improved specificity to fibrin and increased resistance to degradation by endogenous enzymes plasminogen activator inhibitor-1(PAI-1), resulting in longer half-life (1, 19, 21, 25). With these modifications, TNK has a faster rate of administration, greater efficacy, lower bleed risk, and faster door-to-needle time (DTN). More recent studies have shown that TNK may have potential advantages over Alteplase for AIS (12, 13, 15, 16, 17, 18, 27). There have been several large, randomized trials evaluating TNK for the treatment of AIS (2, 4, 5, 6, 28). The NOR-TEST study was one of the first large prospective studies with 1,100 patients in combined study arms (2). The trial demonstrated TNK had favorable 90-day functional outcomes compared to Alteplase and showed similar safety and efficacy (2). The EXTEND-IA TNK study showed that TNK 0.25 mg/kg IV given before thrombectomy was associated with higher rates of reperfusion and better functional outcomes than the standard dose of Alteplase in AIS patients with a large vessel occlusion (LVO), if treated within 4.5 h. of stroke onset (3). The EXTEND-IA TNK part 2 showed that higher TNK doses (0.40 mg/kg) compared with 0.25 mg/kg had no difference in improving cerebral reperfusion prior to endovascular thrombectomy. However, symptomatic ICH occurred in seven patients (4.7%) in the 0.40 mg/kg group compared to two (1.3%) in the 0.25 mg/kg group (4). In 2022, the AcT trial studied 1,577 patients at 22 centers across Canada. It compared TNK 0.25 mg/kg (max 25 mg) to standard alteplase dosing for AIS within 4.5 h (5). The AcT trial showed TNK to be non-inferior to alteplase for the treatment of AIS, with the primary outcome Modified Rankin Score (mRS) of 0–1 being 36.9% in the TNK group and 34.8% in the alteplase group with similar safety outcomes (5). In November 2024, the ATTEST-2 study was published with the largest comparative prospective study to date. This study included 1858 patients from 39 stroke centers in the UK, who were randomized to receive TNK 0.25 mg/kg or alteplase standard of care dose. The study showed TNK was non-inferior to alteplase for 90-day mRS. The study did not demonstrate that TNK was superior for 90-day mRS distribution compared to alteplase. Symptomatic ICH and angioedema were similar between the groups (6). On March 3rd, 2025, the Food and Drug Administration (FDA) approved TNK for AIS based off the AcT trial using fixed dosing based on weight ranges: patients < 60 kg would receive 15 mg Tenecteplase dose (7). The aim of this study was to retrospectively compare alteplase standard dosing to TNK with a specific weight based TNK 0.25 mg/kg Max 25 mg for AIS in clinical practice for patients with LVO and all patients presenting with AIS.

## Methods

### Study design and setting

This is a multicenter retrospective cohort study across 11 primary and comprehensive stroke centers in a single health system in West-Central Florida. We identified adults (≥18 years) with AIS treated with intravenous TNK 0.25 mg/kg (maximum 25 mg) or standard-dose alteplase (0.9 mg/kg, maximum 90 mg) between December 2019 and April 2024 from the electronic health record (Cerner PowerChart). Patients needed to have presented within 4.5 h of last-known-well and met the system criteria for thrombolysis.

### Statistical analysis

Two-sample t-tests compared means; quantile (median) regression compared non-normal continuous and ordinal variables; and two-proportion z-tests (or Fisher’s exact tests when expected counts <5) compared proportions. Multivariable logistic regression estimated adjusted odds ratios (ORs) and 95% confidence intervals (CIs) for binary outcomes (e.g., early reperfusion, 90-day mRS 0–1, 0–2, mortality), and proportional-odds ordinal logistic regression modeled mRS distributions at discharge and 90 days. Models adjusted for treatment group and prespecified covariates (age per 10 years, initial NIHSS, history of cerebrovascular disease, hypertension, atrial fibrillation, diabetes, cardiovascular disease, hyperlipidemia, and smoking). A two-sided *α* of 0.05 defined statistical significance.

The analysis used Minitab 22 for descriptives and two-proportion tests and Stata 15 for binary logistic, ordinal logistic, and quantile regressions.

Sample size calculation was based on the EXTEND-IA TNK trial. Reperfusion occurred in 22% of the TNK group compared to 10% in the alteplase group, with an incidence difference of 12%. Based on this study, it was estimated that a sample size of 200 would achieve 90% power for detecting a 10% difference. A 10% overestimation was added to the 200-sample size estimation to ensure 80% power was obtained. The primary analysis was based on the intention to treat population, and any missing data remained missing. All hypothesis tests used a two-sided significance level set at 0.05.

### Outcomes

The primary outcome (LVO subgroup) was substantial early reperfusion prior to thrombectomy, defined as the restoration of blood flow to >50% of the ischemic territory (mTICI 2b/2c/3) by CTA/MRA or absence of a retrievable thrombus at the initial angiographic assessment. A large vessel occlusion (LVO) was determined at the initial CT/CTA prior to the thrombectomy. Secondary outcomes included door-to-needle time (DTN), NIHSS at 24 h, discharge NIHSS, discharge disposition, 90-day mRS, and safety (symptomatic/asymptomatic ICH by system definitions and angioedema). Safety outcomes for ICH were assessed using the SITS-MOST criteria for symptomatic ICH including subarachnoid hemorrhage within 24 hr of treatment combined with an increase from baseline in NIHSS score of at least 4 points (8).

### Study population

**Figure 1:**
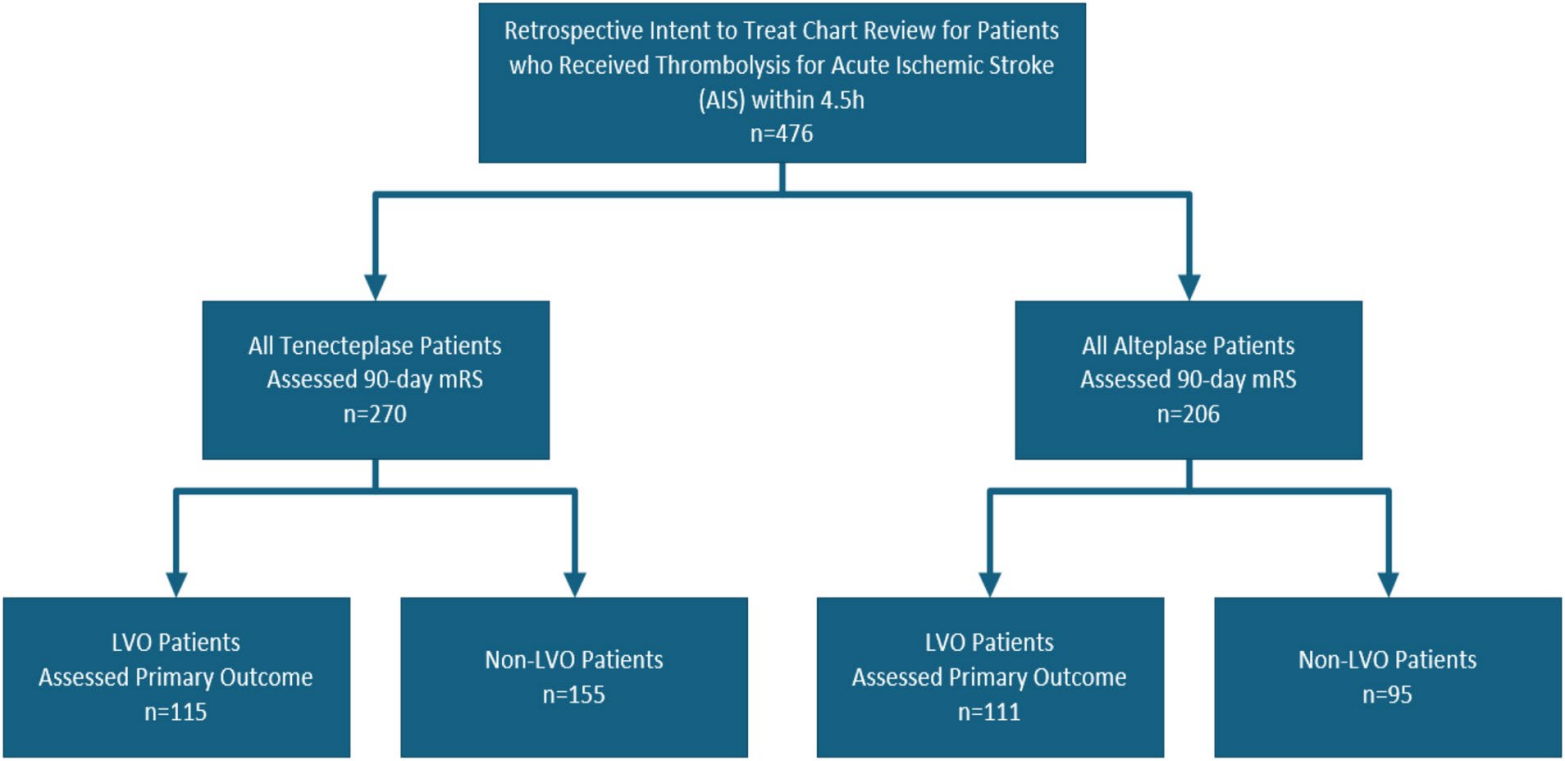
Cohort flow diagram showing sample-size breakdown (All AIS within 4.5 h → Treatment group → LVO status).

The study included 476 AIS patients (TNK *n* = 270, alteplase *n* = 206), of whom 226 had LVO (TNK *n* = 115, alteplase *n* = 111) and 250 had non-LVO stroke (Figure 1). Baseline demographics and initial stroke severity were similar between groups in LVO (median NIHSS 15 vs. 15). Several comorbidities differed in LVO: prior cerebrovascular disease (6.1% TNK vs. 15.3% alteplase), hypertension (73.9% vs. 86.5%), diabetes (31.3% vs. 17.1%), and cardiovascular disease (20.9% vs. 34.2%). Occlusion patterns were broadly comparable, with M1 most common (Table 1). Across all treated patients (TNK, *n* = 270; alteplase, *n* = 206), baseline characteristics were broadly similar between groups (Supplementary Tables 1, 5).

**Table 1:** Baseline characteristics stratified by thrombolytic treatment (LVO patients).

Age and sex	Tenecteplase *n* = 115	Alteplase *n* = 111	Difference (95% CI)	*p* value
Age, y: Mean ±SD	69.2 ± 14.0	71.3 ± 14.8	−2.0 (−5.8 to 1.7)	0.286
Sex, male (%)	71 (61.7%)	64 (57.7%)	4.1% (−8.7 to 16.9%)	0.531

Race	Tenecteplase *n* = 109	Alteplase *n* = 110	Difference (95% CI)	*p* value
Caucasian (%)	83 (76.1%)	93 (84.5%)	−8.4% (−18.9 to 2.1%)	0.116
African American (%)	17 (15.6%)	14 (12.7%)	2.9% (−6.4 to 12.1%)	0.542
Hispanic (%)	1 (0.9%)	0 (0.0%)	0.9% (−0.9 to 2.7%)	0.498
Asian (%)	1 (0.9%)	1 (0.9%)	0.0% (−2.5 to 2.5%)	>0.999
Other (%)	7 (6.4%)	2 (1.8%)	4.6% (−0.6 to 9.8%)	0.101

Type of LVO	Tenecteplase *n* = 115	Alteplase *n* = 111	Difference (95% CI)	*p* value
Basilar (%)	3 (2.6%)	9 (8.1%)	−5.5% (−11.4 to 0.4%)	0.079
ICA (%)	14 (12.2%)	8 (7.2%)	5.0% (−2.7 to 12.6%)	0.204
M1 (%)	35 (30.4%)	47 (42.3%)	−11.9% (−24.4 to 0.6%)	0.061
M2 (%)	23 (20.0%)	16 (14.4%)	5.6% (−4.2 to 15.4%)	0.264
Other MCA (%)	21 (18.3%)	19 (17.1%)	1.1% (−8.8 to 11.1%)	0.822
More than 1 LVO (%)	19 (16.5%)	12 (10.8%)	5.7% (−3.2 to 14.6%)	0.209

Comorbidities	Tenecteplase *n* = 115	Alteplase *n* = 111	Difference (95% CI)	*p* value
History of cerebrovascular disease (%)	7 (6.1%)	17 (15.3%)	−9.2% (−17.2% to −1.2%)	0.024
Hypertension (%)	85 (73.9%)	96 (86.5%)	−12.6% (−22.8% to −2.3%)	0.016
Atrial fibrillation (%)	37 (32.2%)	33 (29.7%)	2.4% (−9.6 to 14.5%)	0.691
Diabetes (%)	36 (31.3%)	19 (17.1%)	14.2% (3.2 to 25.2%)	0.011
Cardiovascular disease (%)	24 (20.9%)	38 (34.2%)	−13.4% (−24.9% to −1.8%)	0.023
Hyperlipidemia (%)	56 (48.7%)	46 (41.4%)	7.3% (−5.7 to 20.2%)	0.272
Smoker (%)	19 (16.5%)	17 (15.3%)	1.2% (−8.3 to 10.7%)	0.804
COVID positive (%)	2 (1.7%)	3 (2.7%)	−1.0% (−4.8 to 2.9%)	0.679

## Results

### Primary outcome (LVO)

Early reperfusion prior to thrombectomy occurred in 17/115 (14.8%) patients treated with TNK vs. 5/111 (4.5%) with alteplase (risk difference 10.3% [95% CI, 2.7–17.8], *p* = 0.008) (Table 2). In multivariable binary logistic regression, TNK was associated with higher odds of early reperfusion compared with alteplase (adjusted OR 3.53, 95% CI 1.20–10.40, *p* = 0.022). Supplementary Figures 1, 3 is a forest plot showing the adjusted odds ratios with 95% confidence intervals from a binary logistic regression of the study’s predictors of early reperfusion prior to thrombectomy (LVO subgroup): treatment group (TNK vs. alteplase), age, initial NIHSS, prior cerebrovascular disease (CVA/TIA), hypertension, atrial fibrillation, diabetes, cardiovascular disease, hyperlipidemia, and smoking status (Table 1).

**Table 2:** Primary outcome (LVO patients).

Primary outcome	Tenecteplase	Alteplase	Difference (95% CI)	*p* value
Substantial reperfusion at initial angiographic assessment no. (%)^*^	17 (14.8%) *n* = 115	5 (4.5%) *n* = 111	10.3% (2.7 to 17.8%)	0.008

* Substantial reperfusion was defined as restoration of blood flow > 50% of the involved ischemic territory (mTICI grade 2b/2c/3) by CT angiogram or MRI or the absence of thrombus on initial angiogram.

### Summary of secondary and safety outcomes

In LVO, 90-day good-to-excellent functional outcome (mRS 0–2) occurred in 47.3% of patients treated with TNK vs. 29.3% with alteplase (*p* = 0.031); excellent outcome (mRS 0–1) was 40.5% vs. 27.6% (*p* = 0.114) (Table 3). In all AIS patients, excellent outcome occurred in 53.2% vs. 45.6% (difference 7.6% [95% CI, −4.0 to 19.2], *p* = 0.199) and good-to-excellent outcome in 61.8% vs. 50.0% (difference 11.8% [95% CI, −0.3 to 23.4], *p* = 0.044). Median 90-day mRS favored TNK (1 [IQR 0–4] vs. 3 [1–5], *p* = 0.046) (Table 4).

**Table 3:** Secondary and safety outcomes (LVO patients).

Secondary outcomes	Tenecteplase	Alteplase	Difference (95% CI)	*p* value
mRS score 0–1 at 90 days (excellent outcome) (%)^†^	30 (40.5%) *n* = 74	16 (27.6%) *n* = 58	13.0% (−3.1 to 29.0%)	0.114
mRS score 0–2 at 90 days (good-to-excellent outcome) (%)^†^	35 (47.3%) *n* = 74	17 (29.3%) *n* = 58	18.0% (1.7 to 34.3%)	0.031
Initial NIHSS score: median (IQR)^¶^	15 (7–22) *n* = 115	15 (8–20) *n* = 111	0 (−3.5 to 3.5)	>0.999
NIHSS score at 24 h. post thrombolytic administration: median (IQR)^¶^	6 (1–15) *n* = 112	7 (2–15) *n* = 108	−1 (−4.9 to 2.9)	0.616
Discharge NIHSS score: median (IQR)^¶^	3 (0–10) *n* = 95	2 (0–10) *n* = 95	1 (−1.3 to 3.3)	0.400
Discharged home or rehab (%)	95 (84.1%) *n* = 113	88 (80.0%) *n* = 110	4.1% (−6.0 to 14.1%)	0.428
Discharged home (%)	51 (45.1%) *n* = 113	39 (35.5%) *n* = 110	9.7% (−3.1 to 22.5%)	0.139
90-day mortality (mRS) (%)^†^	17 (23.0%) *n* = 74	25 (43.1%) *n* = 58	−20.1% (−36.1% to −4.2%)	0.013

Safety outcomes	Tenecteplase	Alteplase	Difference (95% CI)	*p* value
Symptomatic ICH post administration within 24 h. (%)^**^	10 (8.7%) *n* = 115	17 (15.3%) *n* = 111	−6.6% (−15.1 to 1.8%)	0.125
Asymptomatic ICH (%)	5 (4.3%) *n* = 115	10 (9.0%) *n* = 111	−4.7% (−11.2 to 1.8%)	0.160
Angioedema (%)	1 (0.9%) *n* = 115	0 (0.0%) *n* = 111	0.9% (−0.8 to 2.6%)	>0.999

^†^Scores on the modified Rankin scale range from 0 (no neurological deficit) to 6 (death). Functional independent outcome was defined as a modified Rankin scale of 0–2. An excellent outcome was defined as a modified Rankin scale of 0 or 1.

^¶^Scores on the National Institutes of Health Stroke Scale (NIHSS), a standardized neurological examination, range from 0 (normal function) to 42 (deaths), with lower scores indicating less severe stroke.

** Symptomatic intracerebral hemorrhage was defined as a large parenchymal hematoma (blood clot occupying > 30% of the infarct volume with mass effect) and an increase of 4 points or more on NIHSS score.

**Table 4:** Secondary and safety outcomes (all stroke patients: LVO and non-LVO).

Secondary outcomes	Tenecteplase	Alteplase	Difference (95% CI)	*p* value
Door-to-needle time, mins: median (IQR)	34 (27–44) *n* = 178	45 (36–53) *n* = 110	−11 (−14.4 to −7.6)	<0.001
90-day mRS: median (IQR)	1 (0–4) *n* = 186	3 (1–5) *n* = 114	0 (−1 to 0)	0.046
mRS score 0–1 at 90 days (excellent outcome) (%)^†^	99 (53.2%) *n* = 186	52 (45.6%) *n* = 114	7.6% (−4.0 to 19.2%)	0.199
mRS score 0–2 at 90 days (good-to-excellent outcome) (%)^†^	115 (61.8%) *n* = 186	57 (50.0%) *n* = 114	11.8% (−0.3 to 23.4%)	0.044
mRS score 6 at 90 days (%)	24 (12.9%) *n* = 186	27 (23.7%) *n* = 114	−10.8% (−20.0% to −1.6%)	0.021
Discharged home or rehab (%)	241 (89.9%) *n* = 268	178 (86.8%) *n* = 205	3.1% (−2.8 to 9.0%)	0.301
Discharged home (%)	156 (58.2%) *n* = 268	100 (48.8%) *n* = 205	9.4% (0.4 to 18.5%)	0.041
90-day mortality (mRS) (%)^†^	24 (12.9%) *n* = 186	27 (23.7%) *n* = 114	−10.8% (−20.0% to −1.60%)	0.021

Safety outcomes	Tenecteplase	Alteplase	Difference (95% CI)	*p* value
Symptomatic ICH post administration within 24 h. (%)^**^	15 (5.6%) *n* = 270	19 (9.2%) *n* = 206	−3.7% (−8.5 to 1.1%)	0.135
Asymptomatic ICH post administration within 24 h. (%)	8 (3.0%) *n* = 270	12 (5.8%) *n* = 206	−2.9% (−6.6 to 0.9%)	0.138
Combined ICH post administration 24 h. (%)	23 (8.5%) *n* = 270	31 (15.0%) *n* = 206	−6.5% (−12.4% to −0.6%)	0.030
Mortality Symptomatic ICH discharge (%)^**^	6 (2.2%) *n* = 270	13 (6.3%) *n* = 206	−4.1% (−7.8 to −0.3%)	0.033
Angioedema (%)	2 (0.8%) *n* = 266	0 (0.0%) *n* = 205	0.8% (−0.3 to 1.8%)	0.507

^†^Scores on the modified Rankin scale range from 0 (no neurological deficit) to 6 (death). Functional independent outcome was defined as a modified Rankin scale of 0–2. An excellent outcome was defined as a modified Rankin scale of 0 or 1.

NIHSS score at 24 h was lower with TNK (median 6 [IQR = 1–15]) compared to alteplase (7 [2–15]) for the LVO group, but the difference was not statistically significant (*p* = 0.616). Similarly, discharge NIHSS score was higher with TNK (median 3 [IQR = 0–10]) compared to alteplase (2 [0–10]) for the LVO group, but the difference was not statistically significant (*p* = 0.400) (Table 3). While there was a higher percentage of patients discharged home with TNK compared to alteplase, 45.1% versus 35.5%, respectively, for the LVO group, there was not a statistically significant difference. When evaluating the 90-day mRS scale distribution between the two LVO groups, more patients in the TNK group had good to excellent outcomes compared to the alteplase group, 47.3% versus 29.3%, respectively (*p* = 0.031) (Table 3). Patients with excellent outcomes at 90 days occurred in 40.5% of patients treated with TNK compared to 27.6% with alteplase for the LVO group (*p* = 0.114) (Table 3).

In LVO, 90-day mortality (mRS) was statistically lower with TNK compared to alteplase, 23.0% versus 43.1%, respectively (difference −20.1% [95% CI, −36.1% to −4.2%], *p* = 0.013). Also, in LVO, symptomatic ICH occurred in 8.7% of patients treated with TNK vs. 15.3% with alteplase (*p* = 0.125); asymptomatic ICH 4.3% vs. 9.0% (*p* = 0.160). Across all AIS patients, combined ICH (symptomatic and asymptomatic) was less frequent with TNK (8.5% vs. 15.0%, *p* = 0.030), and mortality among symptomatic ICH at discharge was lower (2.2% vs. 6.3%, *p* = 0.033). Angioedema was uncommon (0.8% TNK vs. 0%) (Table 3).

Symptomatic ICH occurred in 5.6% of all patients who received TNK and 9.2% in the alteplase group (difference −3.7% [95% CI, −8.5 to 1.1%], *p* = 0.135). Asymptomatic ICH occurred in 3.0% of the TNK group compared to 5.8% of the alteplase group (absolute difference, −2.9%; 95% CI, −6.6 to 0.9%; *p* = 0.138) for all patients. There was no statistical difference in bleeding complications between groups for LVO, however, there was a statistical difference between TNK and alteplase for the combined groups, 8.5% for TNK versus 15.0% for alteplase, (difference −6.5% [95% CI, −12.4% to −0.6%], *p* = 0.030) (Table 4). In addition, when evaluating all patients who received thrombolytics, there was a statistical difference in symptomatic ICH mortality at discharge between the groups with 6 patients in the TNK group (2.2%) compared to 13 patients in the alteplase group (6.3%) (difference −4.1% [95% CI, −7.8% to −0.3%], *p* = 0.033) (Table 4). Angioedema after administration occurred in 2 patients in the TNK group (0.8%) and none in the alteplase group (0.0%) but was not statistically significant (difference 0.8% [95% CI, −0.3 to 1.8%], *p* = 0.507). The 90-day mRS for mortality for all patients occurred in 12.9% of the TNK group and 23.7% of the alteplase group (difference −10.8% [95% CI, −20.0% to −1.60%], *p* = 0.021) (Table 4).

**Figure 2:**
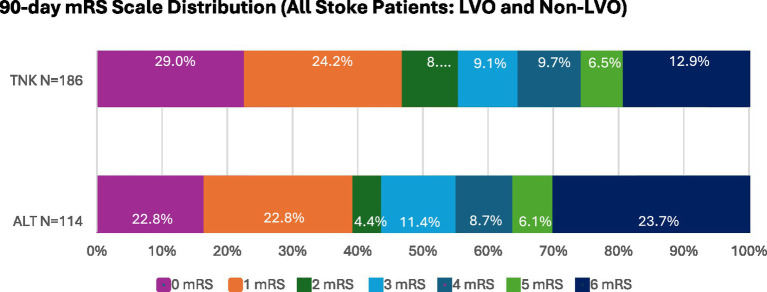
Modified Rankin scale scores at 90 days in the intention to treat. Shown is the scale distribution of the modified Rankin scale scores at 90 days. Scores range from 0 to 6, with 0 indicating no neurological deficit. 1 no clinical significant disability, 2 slight disability (able to handle own affairs without assistance but unable to carry out all previous activities), 3 moderate disability requiring some help (e.g. with shopping, cleaning, and finances but able to walk unassisted), 4 moderately severe disability (unable to attend to bodily needs without assistance and unable to walk unassisted), 5 severe disability (requiring constant nursing care and attention), and 6 death. Patients in the TNK group had a median score of 1, as compared with a median score of 3 among patients in the Alteplase group (95% CI: −1 to 0, *p*=0.046).

Among all AIS patients, DTN was shorter with TNK (median 34 [IQR 27–44] minutes) than with alteplase (45 [36–53] minutes), an11-minute difference (95% CI, −14.4 to −7.6; *p* < 0.001) (Table 4). Evaluation of the scale distribution of 90-day mRS in all patients who received thrombolytics showed patients with excellent outcome (mRS 0–1) occurred in 53.2% of all patients that received TNK versus 45.6% for the alteplase group (difference 7.6% [95% CI, −4.0 to 19.2%], *p* = 0.199). Good-to-excellent outcome (mRS 0–2) occurred in 61.8% in all patients who received TNK and 50.0% in the alteplase group (statistically significant difference 11.8% [95% CI, −0.3 to 23.4%], *p* = 0.044) (Table 4). In Figure 2 listed below shows the 90 day modified rankin score distribution between TNK and alteplase favoring TNK. There was a statistical difference in all the patients who were discharged home with 58.2% in the TNK group versus 48.8% in the alteplase group (difference 9.4% [95% CI, 0.4 to 18.5%], *p* = 0.041) (Table 4).

### Adjusted associations for 90-day good outcome (LVO subgroup)

In the LVO subgroup’s logistic model for good outcome (mRS 0–2), treatment group was not a significant independent predictor (TNK vs. alteplase: OR 1.69 [95% CI, 0.71–4.00], *p* = 0.236). Lower initial stroke severity and younger age were associated with higher odds of a good outcome (initial NIHSS per point: OR 0.91 [95% CI, 0.85–0.96], *p* = 0.001; age per 10 years: OR 0.73 [95% CI, 0.53–1.00], *p* = 0.048). Hyperlipidemia was positively associated with 90 day mRS score for good outcome (mRS 0-2) (OR 2.55 [95% CI, 1.08-6.03], *p* = 0.033. Supplementary Figures 3, 5 is a forest plot showing the adjusted odds ratios with 95% confidence intervals based on binary logistic regression of the study’s predictors of a 90-day good outcome: treatment group (TNK vs. alteplase), age, initial NIHSS, prior cerebrovascular disease (CVA/TIA), hypertension, atrial fibrillation, diabetes, cardiovascular disease, hyperlipidemia, and smoking status.

### Adjusted associations for 90-day ordinal outcome (LVO subgroup)

In the LVO subgroup’s logistic model for ordinal outcome mRS, treatment group was not a significant independent predictor (TNK vs. alteplase: OR 0.58 [95% CI, 0.29–1.17], *p* = 0.129). Higher initial stroke severity and older age were associated with higher odds of higher 90-day mRS ordinal outcomes (initial NIHSS per point: OR 1.09 [95% CI, 1.05–1.14], *p* < 0.001; age per 10 years: OR 1.43 [95% CI, 1.12–1.82], *p* = 0.004). Hyperlipidemia was negatively associated with higher 90-day mRS ordinal outcomes (OR 0.44 [95% CI, 1.08–6.03], *p* = 0.033). Supplementary Figures 3, 5 is a forest plot showing the adjusted odds ratios with 95% confidence intervals based on binary logistic regression of the study’s predictors of 90-day mRS ordinal outcome: treatment group (TNK vs. alteplase), age, initial NIHSS, prior cerebrovascular disease (CVA/TIA), hypertension, atrial fibrillation, diabetes, cardiovascular disease, hyperlipidemia, and smoking status.

### Adjusted associations for mortality related to a bleed outcome (LVO subgroup)

In the LVO subgroup logistic model for bleeding-related mortality, treatment group was not a significant independent predictor (TNK vs. alteplase: OR 0.25; 95% CI, 0.04–1.52; *p* = 0.132). Hyperlipidemia was negatively associated with higher odds of mortality related to a bleed (OR 0.14 [95% CI, 0.03–0.76], *p* = 0.023). Supplementary Figures 3, 7 is a forest plot showing the adjusted odds ratios with 95% confidence intervals based on a binary logistic regression of the study’s predictors of bleeding-related mortality related: treatment group (TNK vs. alteplase), age, initial NIHSS, prior cerebrovascular disease (CVA/TIA), hypertension, atrial fibrillation, diabetes, cardiovascular disease, hyperlipidemia, and smoking status.

## Discussion

In a large integrated community-hospital health system, TNK 0.25 mg/kg was associated with significantly higher early reperfusion prior to thrombectomy among LVO patients and shorter DTN across all AIS patients, without excess bleeding. Functional outcomes favored TNK in several analyses (including LVO mRS 0–2 and median 90-day mRS), while adjusted odds of excellent outcome did not differ significantly between agents after accounting for key covariates (age, stroke severity, and comorbidities). Findings align with randomized evidence supporting TNK as an effective alternative to alteplase and suggest potential workflow advantages related to single-bolus administration.

Also, across additional multivariable models, TNK consistently achieved higher rates of early reperfusion while not demonstrating independent superiority on 90-day mRS after adjusting for age and initial NIHSS. Age and baseline stroke severity were the most robust determinants of functional outcomes and mortality. The repeated association between hyperlipidemia and favorable outcomes likely reflects confounding by statin exposure or overall cardiovascular risk management rather than a causal protective effect; nevertheless, the signal was directionally consistent across models. Taken together with shorter door-to-needle times and comparable safety, these data support the clinical and operational advantages of TNK dosed at 0.25 mg/kg in real-world community hospital LVO and AIS care.

## Conclusion

This retrospective observational study reinforces current published evidence that TNK at 0.25 mg/kg IV (maximum 25 mg) is a safe and effective alternative to standard-dose alteplase for AIS. The primary outcome in this observational study did show similar results to the EXTEND-IA study, with TNK resulting in a 10.3% reperfusion rate difference compared to Alteplase for LVO prior to thrombectomy which was statistically significant *p* = 0.008. The number needed to treat (NNT) to see 50% reperfusion prior to thrombectomy in the TNK group was 7 in this study. This was slightly higher than in the EXTEND IA trial, where it was 5 (3). Also, in the adjusted logistic regression model, the odds of achieving reperfusion prior to thrombectomy were 3.53 times higher with TNK than with alteplase (OR = 3.53 [95% CI, 1.20 to 10.40], *p* = 0.022) (Supplementary Figures 1, 3), resulting in an absolute improvement of approximately 10 percentage points (model-based average marginal effect of 9.7 percentage points) which closely corresponds to the observed 10.3% absolute difference. These findings are consistent with EXTEND-IA and reinforce that 0.25 mg/kg IV (max 25 mg) is a safe, efficacious dose (3, 28).

This study also showed that TNK has similar safety outcomes to alteplase for AIS. Operationally, TNK reduced door-to-needle time by 11 min (*p* < 0.001) (Table 4), which is clinically meaningful given established time-outcome relationships and may have contributed to favorable secondary outcomes (e.g., higher 90-day mRS 0–2 in LVO). This could be due to the ease of administration of TNK compared to alteplase. The AcT trial sub-analysis showed that, for every 30-min reduction in onset to needle time, an additional 2% of patients achieved an excellent outcome. For every 60 min reduction in door-to-needle time, an additional 1% of patients achieved an excellent outcome (9). The ease of administration allows for smoother transition during a transfer to a comprehensive center for thrombectomy. Although this retrospective design and the missing 90-day mRS data introduce limitations, the study reflects diverse, real-world community hospital utilization with safety similar to alteplase.

The FDA approved Tenecteplase (TNKase®) for the treatment of AIS on March 3^rd^ (10). The recommended package insert dosing was based off the AcT study dosing algorithm, which utilizes weight ranges rounding to the nearest 2.5 mg (5). Patients less than 60 kg would receive 15 mg on the low end and there is a maximum dose of 25 mg on the high end. The 0.25 mg/kg TNK IV dose was not included in the package insert and is the dose currently recommended in the 2022 European stroke guidelines. The AcT study conclusion was that 0.25 mg/kg IV TNK is a reasonable alternative to alteplase for all patients presenting with AIS who meet standard criteria for thrombolysis (5). The NOR-TEST-2 trial evaluated 0.4 mg/kg IV TNK and was terminated early based on overall ICH bleeds at 21% (11). Based on the approved dosing label, extremely low-weight patients could approach 0.4 mg/kg weight based dose with the fixed 15 mg TNK dose. The AcT study did not specify the number of patients < 60 kg in their results to validate the safety of the 15 mg dose in extremely low-weight patients included in the package insert dosing for TNK. This study provides additional evidence to support the 0.25 mg/kg IV TNK dose. The dosing protocol used in this trial was based on actual body weights dosed at 0.25 mg/kg IV with max of 25 mg rounded to the nearest 1 mg (see Appendix 1). The ATTEST-2 study also used weight-based dosing at 0.25 mg/kg IV max 25 mg rounding to the nearest 0.5 mg. In the post-hoc analysis evaluating safety with weight < 60 kg in Supplementary Tables 2, 6, our study included 21 patients in this low weight category who received TNK 0.25 mg/kg, with the lowest dose given being 13 mg. One patient in the post-hoc analysis (4.8%) developed a symptomatic bleed, as did two patients with asymptomatic ICH within 24 h. of administration. The post-hoc analysis in the < 60 kg TNK and alteplase groups had similar demographic (Supplementary Figures 3, 7). Further studies are needed to evaluate TNK in the lower weight population with 15 mg fixed Tenecteplase dose for patients < 60 kg. The data from this study, coupled with easier administration and faster treatment times, further supports TNK 0.25 mg/kg as a preferred thrombolytic for eligible AIS patients.

## Data Availability

The raw data supporting the conclusions of this article will be made available by the authors, without undue reservation.
