# Supplementary material for: The evaluation of Tenecteplase for the treatment of ischemic stroke (real-world data)
Source: Front Neurol. 2026 Jul 7;17:1869006. doi: 10.3389/fneur.2026.1869006 (PMC13387016; doi:10.3389/fneur.2026.1869006)
Supplement: Supplementary file 1 [file Supplementary_File_1.pdf]

Supplement

Supplement Table 1. Demographics and Sample Characteristics (All Stroke Patients: LVO and Non-LVO)

Demographics and Sample Characteristics	Tenecteplase n=270	Alteplase n=206	Difference (95% CI)	P value
Age, y: Mean ±SD	68.2 ±14.6	70.0 ±15.4	-1.8 (-4.5 to 1.0)	0.210
Sex, Male (%)	145 (53.7%)	106 (51.5%)	2.2% (-6.8% to 11.3%)	0.627
Initial NIHSS score: Median (IQR)*	8 (4-15)	9 (5-16)	-1 (-2.8 to 0.8)	0.282
Race	Tenecteplase n=263	Alteplase n=204	Difference (95% CI)	P value
Caucasian (%)	212 (80.6%)	174 (85.3%)	-4.7% (-11.5% to 2.1%)	0.178
African American (%)	33 (12.5%)	23 (11.3%)	1.3% (-4.6% to 7.2%)	0.673
Hispanic (%)	3 (1.1%)	1 (0.5%)	0.7% (-1.0% to 2.2%)	0.635
Asian (%)	3 (1.1%)	2 (1.0%)	0.2% (-1.7% to 2.0%)	>0.999
Other (%)	11 (4.2%)	4 (2.0%)	2.2% (-0.9% to 5.3%)	0.198
Comorbidities	Tenecteplase n=253	Alteplase n=202	Difference (95% CI)	P value
History Cerebrovascular Disease (%)	23 (9.1%)	26 (12.9%)	-3.8% (-9.6% to 2.0%)	0.203
Hypertension (%)	204 (80.6%)	165 (81.7%)	-1.1% (-8.3% to 6.2%)	0.776
Atrial Fibrillation (%)	50 (19.8%)	49 (24.3%)	-4.5% (-12.2% to 3.2%)	0.252
Diabetes (%)	77 (30.4%)	43 (21.3%)	9.1% (1.1% to 17.1%)	0.025
Cardiovascular Disease (%)	52 (20.6%)	68 (33.7%)	-13.1% (-21.3% to -4.9%)	0.002
Hyperlipidemia (%)	123 (48.6%)	89 (44.1%)	4.6% (-4.7% to 13.8%)	0.332
Smoker (%)	58 (22.9%)	34 (16.8%)	6.1% (-1.2% to 13.4%)	0.102
COVID (%)	9 (3.6%)	4 (2.0%)	1.6% (-1.4% to 4.6%)	0.402

*Scores on the National Institutes of Health Stroke Scale (NIHSS), a standardized neurological examination, range from 0 (normal function) to 42 (deaths), with lower scores indicating less severe stroke.

Post Hoc Safety Analysis for Patients with weight < 60 kg

Supplement Table 2. Secondary and Safety Outcomes (All Stroke Patients with weight < 60 kg: LVO and Non-LVO)

Safety Outcomes	Tenecteplase	Alteplase	Difference (95% CI)	P value
Symptomatic ICH post administration within 24 hrs. (%)**	1 (4.8%) n=21	0 (0.0%) n=15	4.8% (-4.3% to 13.9%)	>0.999
Asymptomatic ICH post administration within 24hrs. (%)	2 (9.5%) n=21	2 (13.3%) n=15	-3.8% (-25.1% to 17.5%)	>0.999
Angioedema (%)	0 (0.0%) n=21	0 (0.0%) n=15	0.0% (Not estimable – no events in either group)	>0.999

** Symptomatic intracerebral hemorrhage was defined as a large parenchymal hematoma (blood clot occupying > 30% of the infarct volume with mass effect and an increase of 4 points or more on NIHSS score.

Supplement Figure 1. Adjusted odds ratios for predictors of early reperfusion prior to thrombectomy (LVO subgroup) based on binary logistic regression.

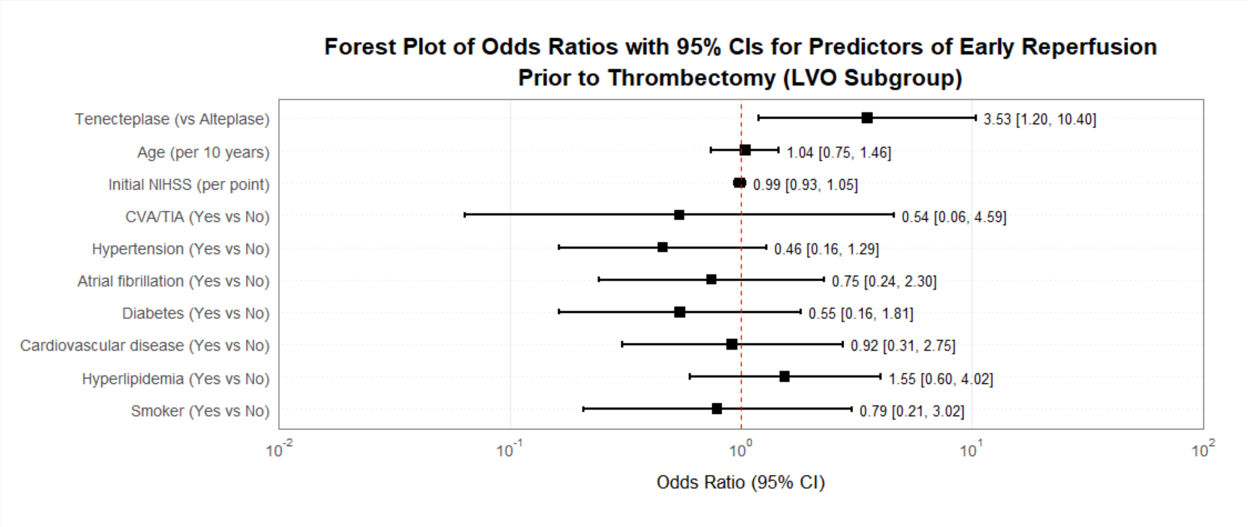

Supplement Figure 2. Adjusted odds ratios for predictors of 90-day excellent outcome (mRS 0–1) (LVO subgroup) based on binary logistic regression.

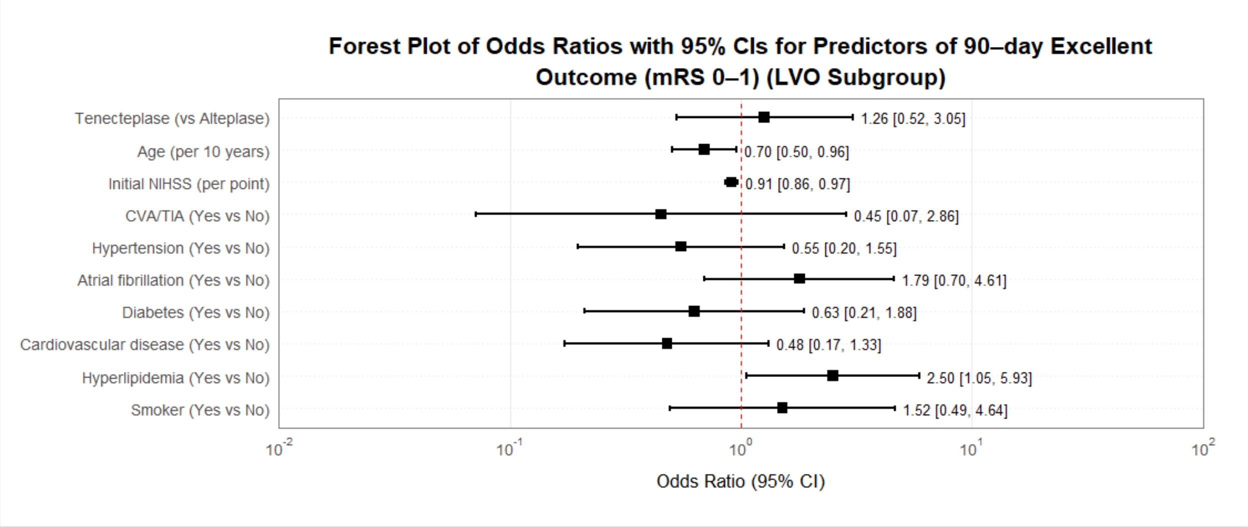

Supplement Figure 3. Adjusted odds ratios for predictors of 90-day good outcome (mRS 0–2) (LVO subgroup) based on binary logistic regression.

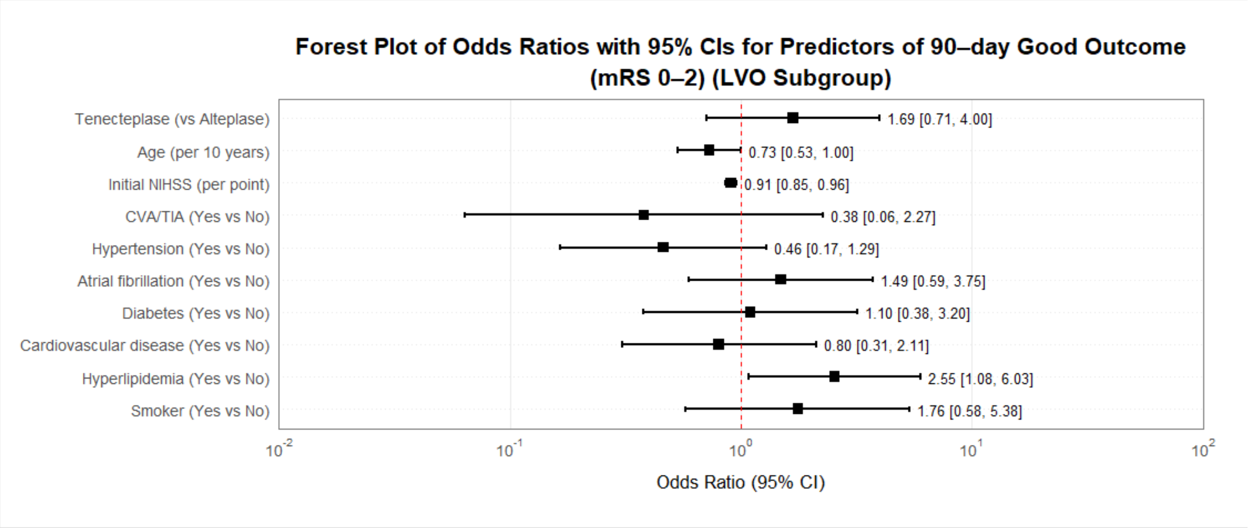

Supplement Figure 4. Adjusted odds ratios for predictors of 90-day mRS ordinal outcome (LVO subgroup) based on proportional-odds ordinal logistic regression

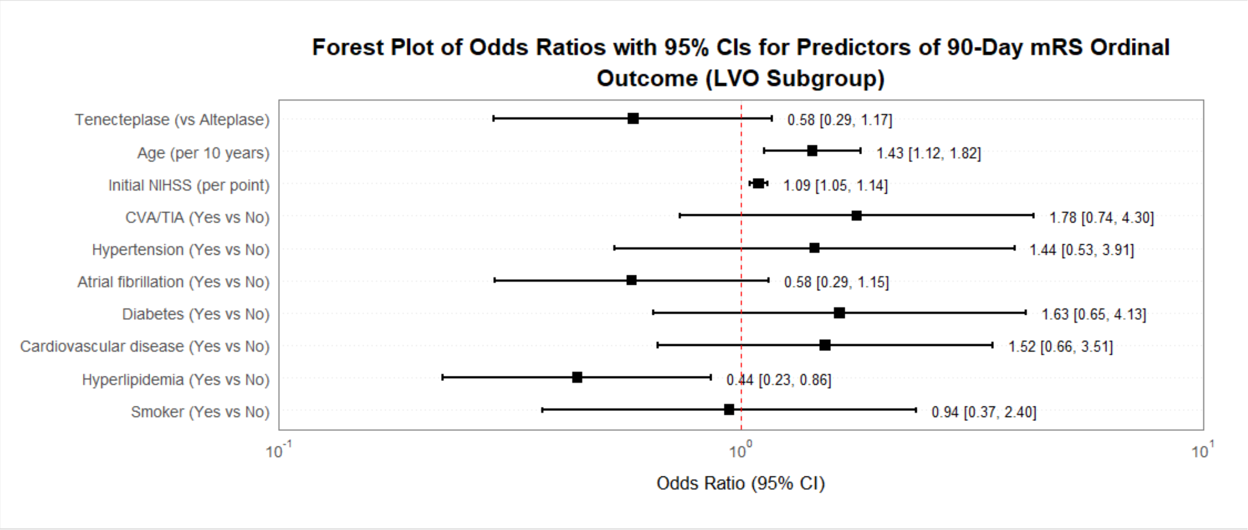

Supplement Figure 5. Adjusted odds ratios for predictors of mortality related to a bleed (LVO subgroup) based on binary logistic regression.

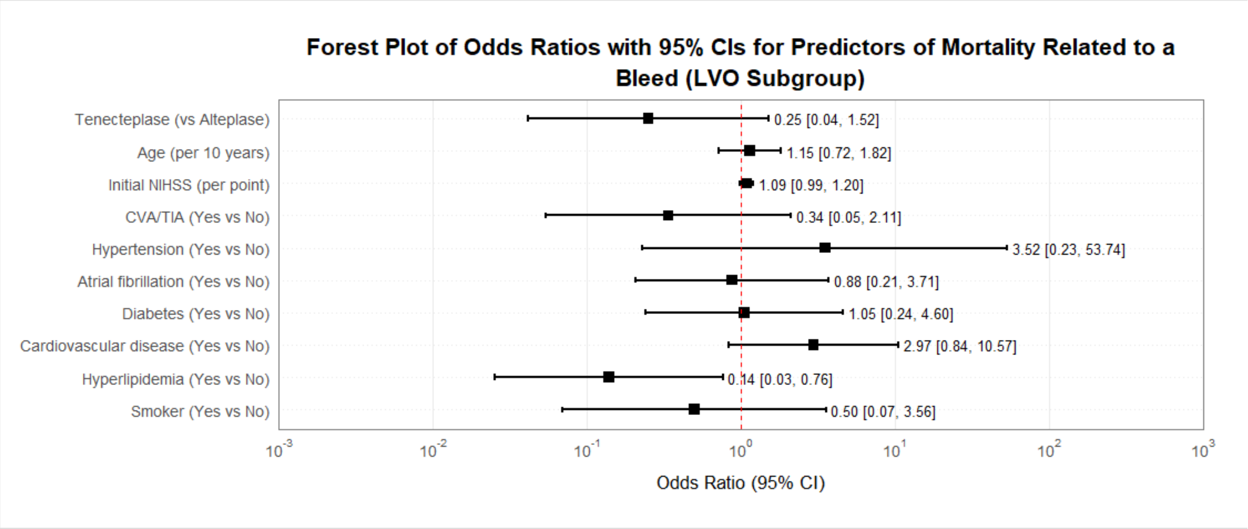

Supplement Figure 6. Adjusted odds ratios for predictors of 90-day all-cause mortality (mRS=6) (LVO subgroup) based on binary logistic regression.

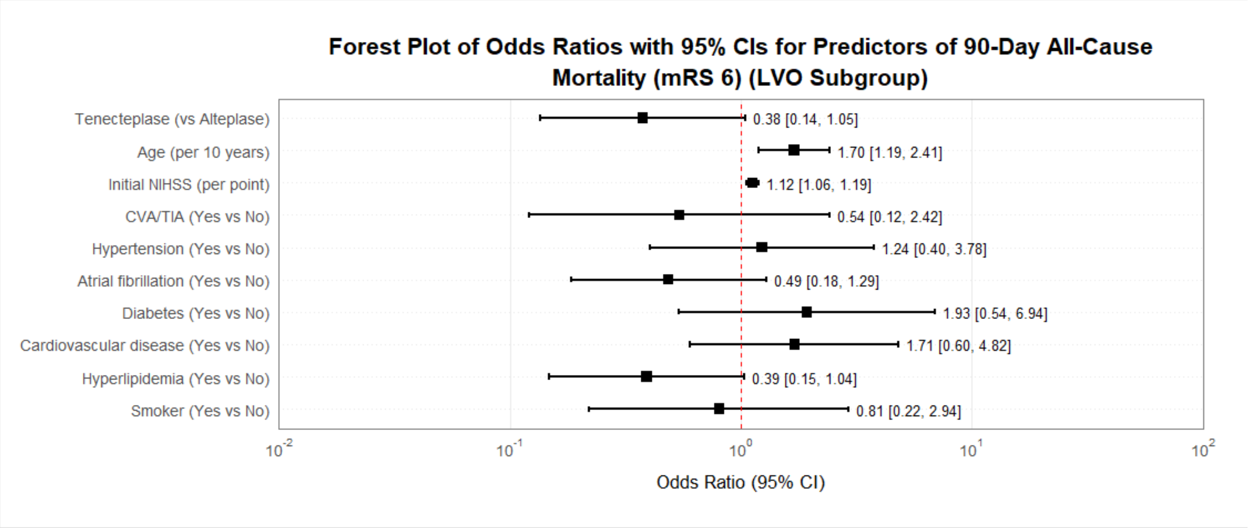

Supplement Figure 7. Predictive margins for early reperfusion prior to thrombectomy by age and treatment group (LVO subgroup) based on binary logistic regression.

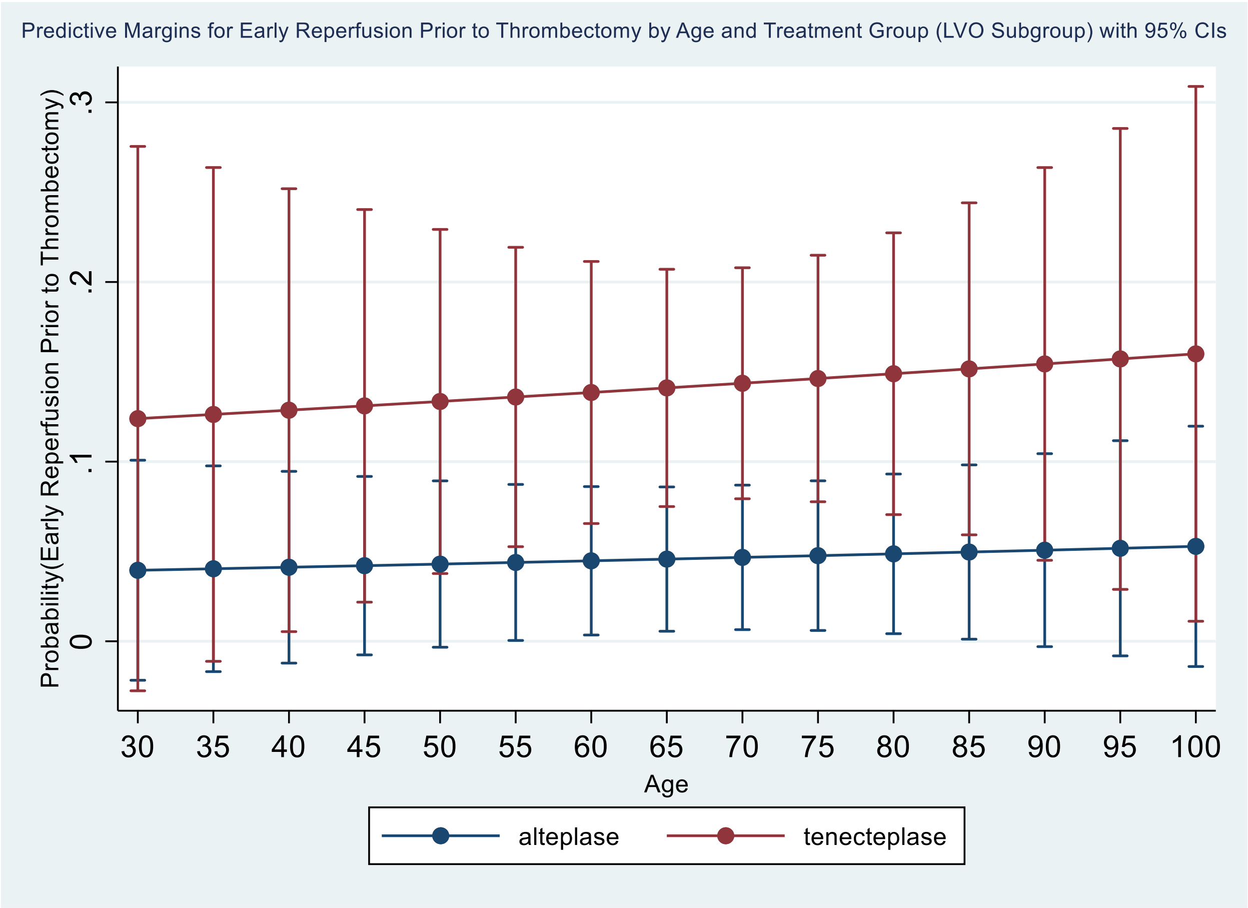

Supplement Figure 8. Predictive margins for early reperfusion without mechanical thrombectomy by initial NIHSS score and treatment group (LVO subgroup) based on binary logistic regression.

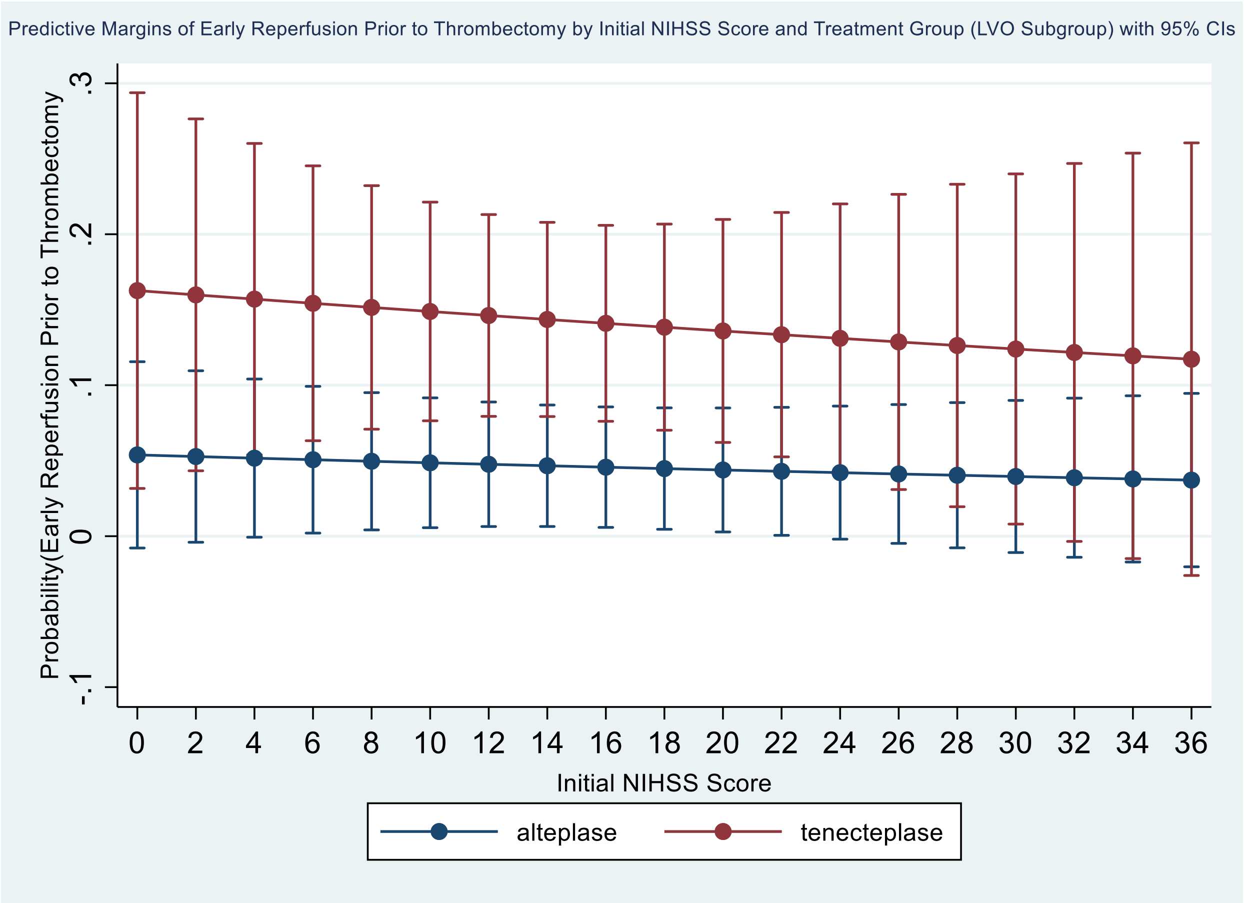

Supplement Figure 9. Predictive margins for 90-day excellent outcome (mRS 0–1) by age and treatment group (LVO subgroup) based on binary logistic regression.

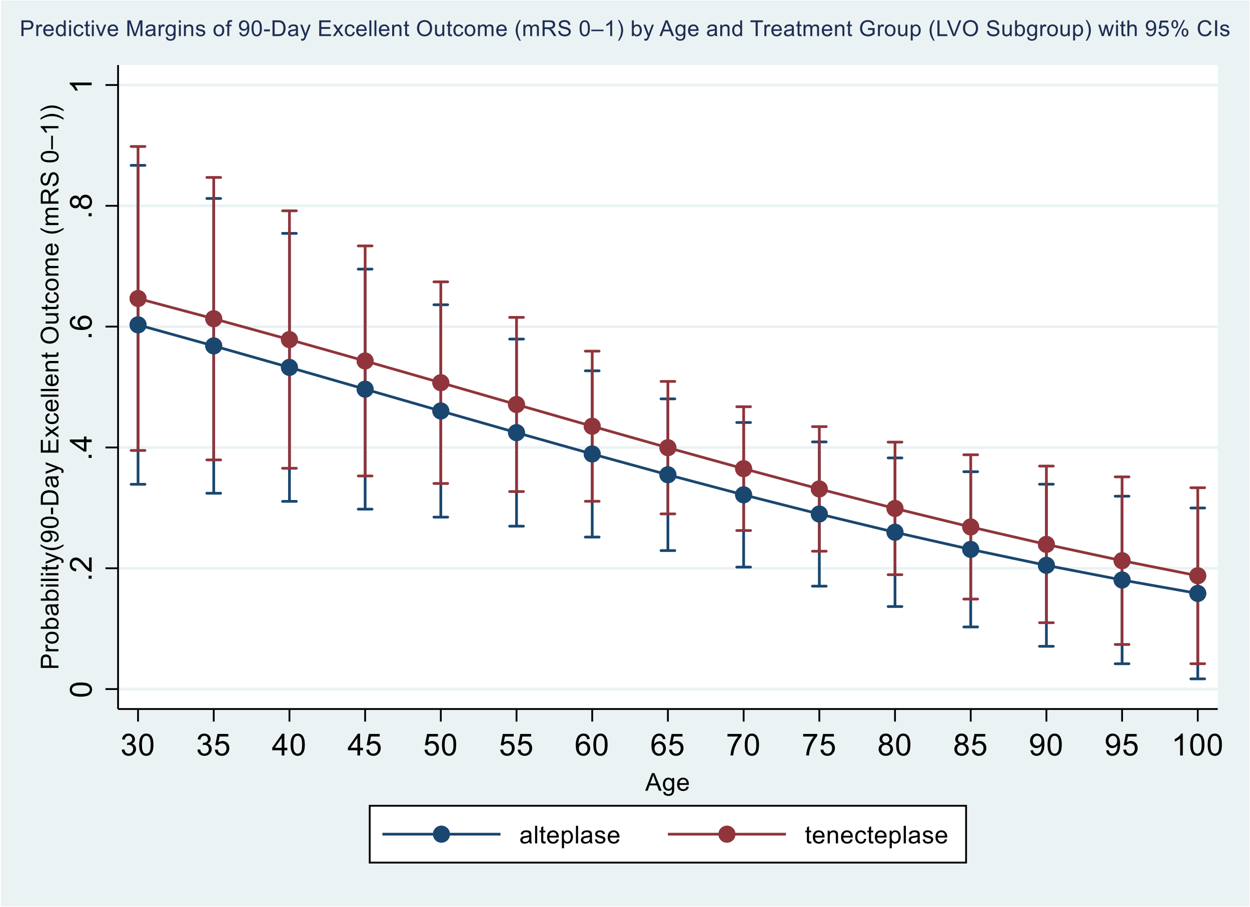

Supplement Figure 10. Predictive margins for 90-day excellent outcome (mRS 0–1) by initial NIHSS score and treatment group (LVO subgroup) based on binary logistic regression.

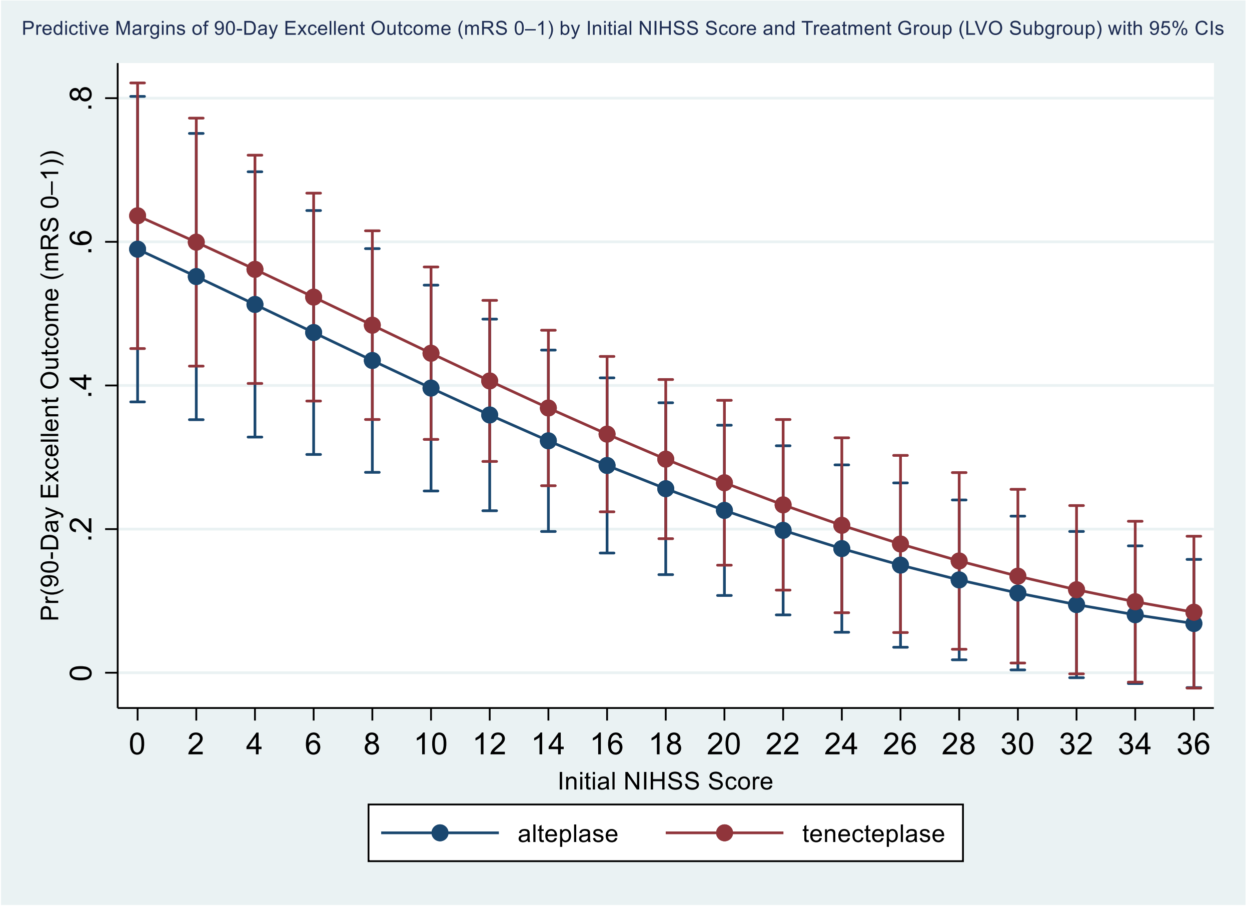

Supplement Figure 11. Predictive margins for 90-day good outcome (mRS 0–2) by age and treatment group (LVO subgroup) based on binary logistic regression.

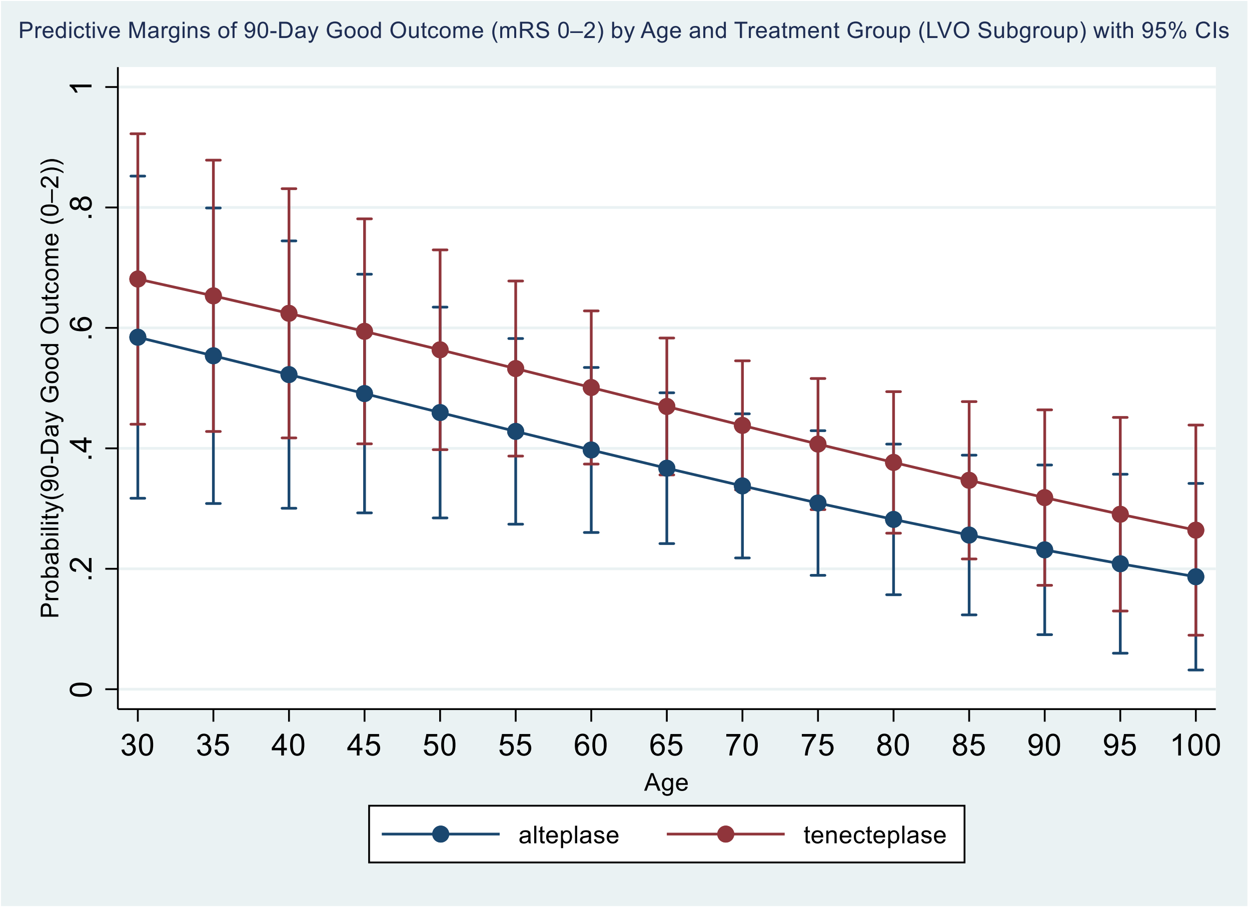

Supplement Figure 12. Predictive margins for 90-day good outcome (mRS 0–2) by initial NIHSS score and treatment group (LVO subgroup) based on binary logistic regression.

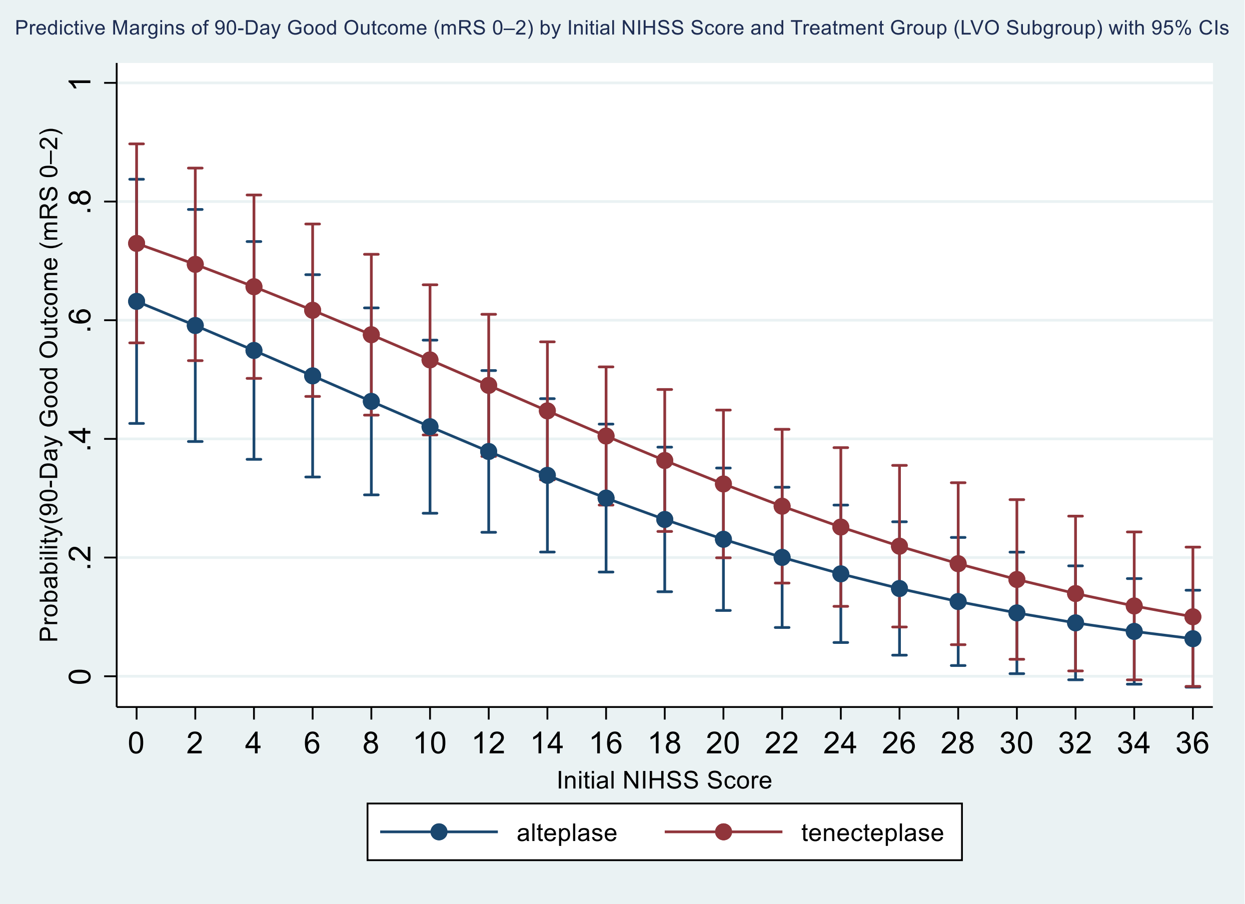

Supplement Figure 13. Predictive margins for 90-day mortality (mRS=6) by age and treatment group (LVO subgroup) based on binary logistic regression.

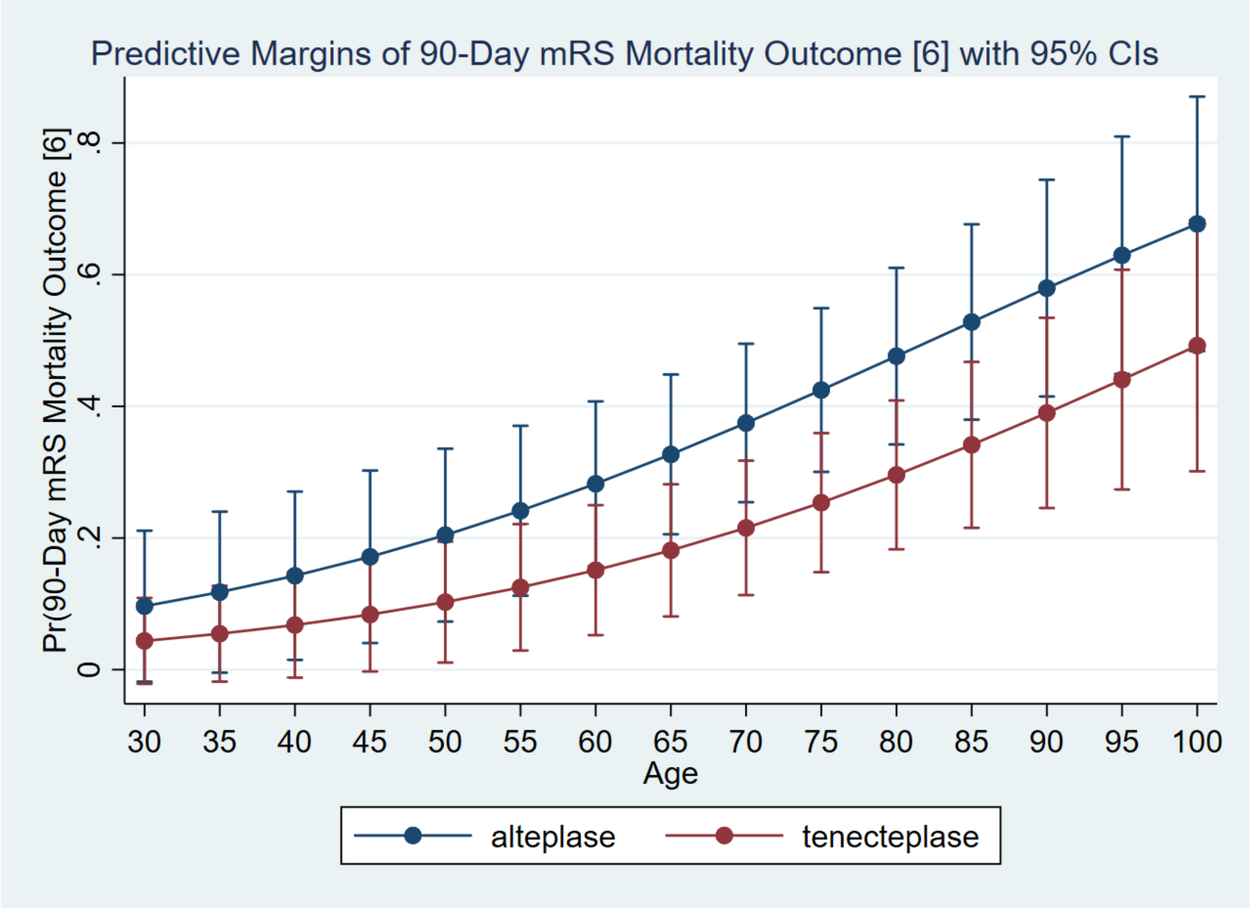

Supplement Figure 14. Predictive margins for 90-day mortality (mRS=6) by initial NIHSS score and treatment group (LVO subgroup) based on binary logistic regression.

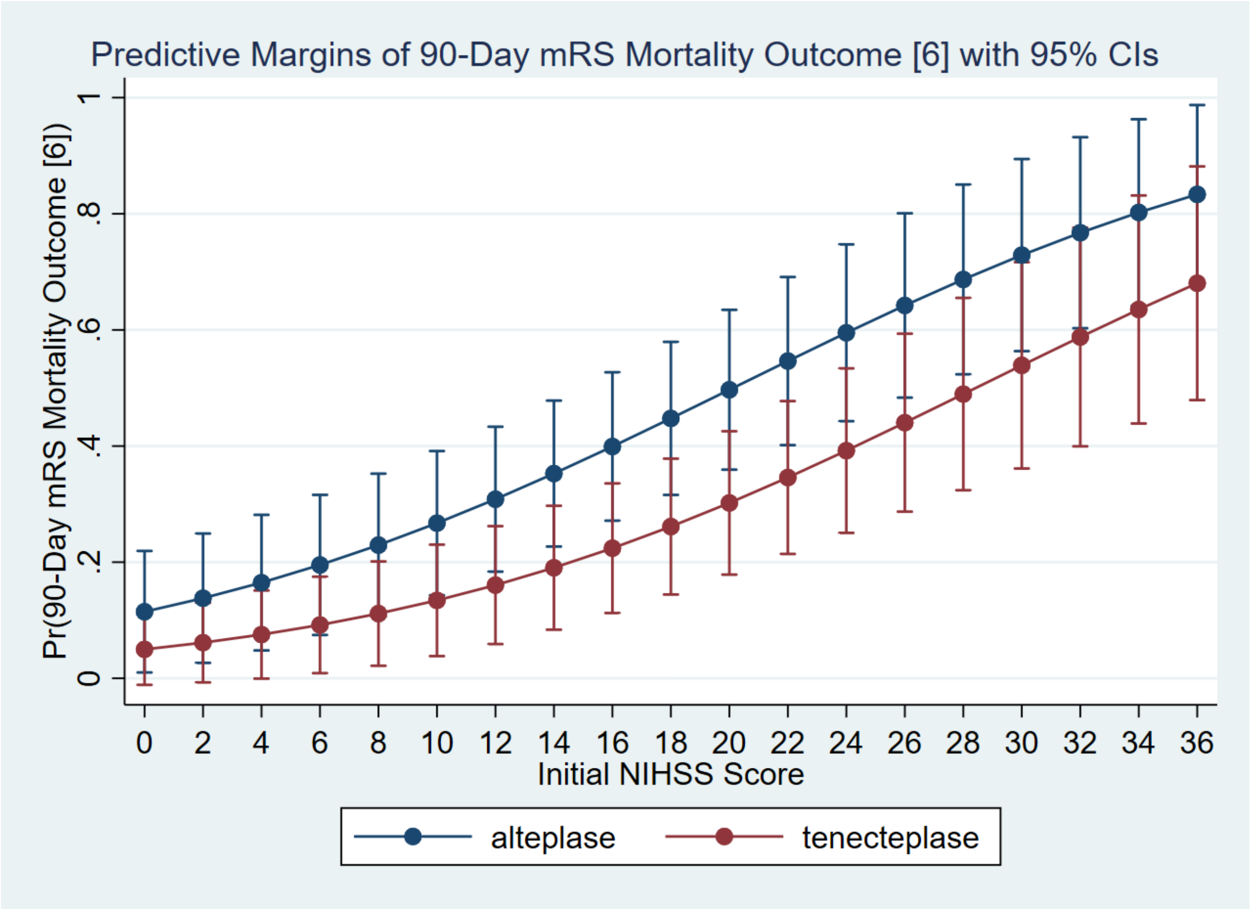

Supplement Table 3

Post Hoc Analysis (Patients <60 kg)

<u>Baseline Characteristics (LVO)</u>

Baseline Characteristics Stratified by Thrombolytic Treatment (LVO Patients)

Age and Sex	Tenecteplase n=9	Alteplase n=5	Difference (95% CI)	P value
Age, y: Mean ±SD	74.8 ±12.3	88.8 ±16.9	-14.0 (-35.1 to 7.1)	0.155
Sex, Male (%)	2 (22.2%)	0 (0.0%)	22.2% (-4.9% to 49.4%)	0.505
**Race**	**Tenecteplase n=8**	**Alteplase n=5**	**Difference (95% CI)**	**P value**
Caucasian (%)	5 (62.5%)	4 (80.0%)	-17.5% (-66.0% to 31.0%)	>0.999
African American (%)	2 (25.0%)	1 (20.0%)	5.0% (-41.1% to 51.1%)	>0.999
Hispanic (%)	0 (0.0%)	0 (0.0%)	0.0% (Not estimable – no events in either group)	>0.999
Asian (%)	1 (12.5%)	0 (0.0%)	12.5% (-10.4% to 35.4%)	>0.999
Other (%)	0 (0.0%)	0 (0.0%)	0.0% (Not estimable – no events in either group)	>0.999
**Type of LVO**	**Tenecteplase n=9**	**Alteplase n=5**	**Difference (95% CI)**	**P value**
Basilar (%)	0 (0.0%)	0 (0.0%)	0.0% (Not estimable – no events in either group)	>0.999
ICA (%)	1 (11.1%)	1 (20.0%)	-8.9% (-49.5% to 31.74%)	>0.999
M1 (%)	2 (22.2%)	3 (60.0%)	-37.8% (-88.6% to 13.0%)	0.266
M2 (%)	5 (55.6%)	1 (20.0%)	35.6% (-12.2% to 83.3%)	0.301
Other MCA (%)	3 (33.3%)	1 (20.0%)	13.3% (-33.3% to 60.0%)	>0.999
More than 1 LVO (%)	2 (22.2%)	1 (20.0%)	2.2% (-42.1% to 46.6%)	>0.999
**Comorbidities**	**Tenecteplase n=9**	**Alteplase n=5**	**Difference (95% CI)**	**P value**
History Cerebrovascular Disease (%)	1 (11.1%)	3 (60.0%)	-48.9% (-96.5% to -1.3%)	0.095
Hypertension (%)	7 (77.8%)	5 (100.0%)	-22.2% (-49.4% to 4.9%)	0.505

Atrial Fibrillation (%)	3 (33.3%)	1 (20.0%)	13.3% (-33.3% to 60.0%)	>0.999
Diabetes (%)	0 (0.0%)	1 (20.0%)	-20.0% (-55.1% to 15.1%)	0.357
Cardiovascular Disease (%)	3 (33.3%)	4 (80.0%)	-46.7% (-93.3% to 0.0%)	0.266
Hyperlipidemia (%)	7 (77.8%)	3 (60.0%)	17.8% (-33.0% to 68.6%)	0.580
Smoker (%)	3 (33.3%)	1 (20.0%)	13.3% (-33.3% to 60.0%)	>0.999
COVID Positive (%)	0 (0.0%)	0 (0.0%)	0.0% (Not estimable – no events in either group)	>0.999

Appendix 1

Tenecteplase – (TNKase) Reconstitution and Dosing for Adult Stroke

Tenecteplase (TNKase) Dose:0.25 mg/kg (**not to exceed 25mg)** Give IV push over 5 seconds (Flush Line before and after with NS flush

- Open the Tenecteplase kit containing a 10 mL syringe, Red hub cannula filling device, TwinPak shield, Blunt plastic cannula, USP Sterile Water for Injection 10 mL, and 50 mg Tenecteplase vial.
- Use aseptic technique by washing hands and using gloves (Swab all vials with alcohol prep prior to reconstitution)
- Remove the flip-caps from 1 vial of Tenecteplase 50mg and 1 vial of 10ml Sterile Water for Injection.
- Flip the cap off both Sterile water for injection 10 ml vial and Tenecteplase vial. Use alcohol pads to wipe the surface of both vials.
- Reconstitution: Remove the syringe assembly from the syringe and set aside. Insert the syringe with the red cap cannula into the sterile water for injection vial. Aseptically withdraw all 10 mL from the SWFI vial into the syringe. Then inject the entire syringe into the Tenecteplase vial using the same red hub cannula directing the stream of water into the powder. Foaming may occur, which is normal. If there are any bubbles, let the vial stand for a minute until bubbles dissipate. **Swirl gently and do not shake**.
- Withdraw the appropriate volume based on chart below **dosed at 0.25 mg/kg with max dose of 25 mg**

Administration of bolus (See Dosing Charts)

- **Step 1: Inspect Solution** - After reconstitution to concentration of mg/ml, inspect solution for particulate matter and discoloration.
- **Step 2: Administer Tenecteplase Dose -** Using a 10 ml Syringe give IV Push over 5 seconds (Follow the dosing chart for appropriate dosing)

Tenecteplase -Tissue Plasminogen Activator (Tenecteplase, TNKase) Weight Dose Chart for Stroke Indication Using 50mg Vial (5mg/ml concentration) Dose 0.25 mg/kg Max Dose 25 mg= 5 mL

Patient Weight		Total Dose 0.25mg/Kg to a Max of 25mg (Rounded to nearest 1 mg)	Total Dose IV Push over 5sec	Patient Weight		Total Dose 0.25mg/Kg to a Max of 25 mg	Total Dose IV Push over 5sec
lbs	Kg	Dose mg	Dose mg=ml	lbs	Kg	Dose mg	Dose mg=ml
79	35.9	9	1.8	103	46.8	12	2.4
80	36.4	9	1.8	104	47.3	12	2.4
81	36.8	9	1.8	105	47.7	12	2.4
82	37.3	9	1.8	106	48.2	12	2.4
83	37.7	9	1.8	107	48.6	12	2.4
84	38.2	10	2	108	49.1	12	2.4
85	38.6	10	2	109	49.5	12	2.4
86	39.1	10	2	110	50.0	12	2.4
87	39.5	10	2	111	50.5	13	2.6
88	40.0	10	2	112	50.9	13	2.6
89	40.5	10	2	113	51.4	13	2.6
90	40.9	10	2	114	51.8	13	2.6
91	41.4	10	2	115	52.3	13	2.6
92	41.8	10	2	116	52.7	13	2.6
93	42.3	11	2.2	117	53.2	13	2.6
94	42.7	11	2.2	118	53.6	13	2.6
95	43.2	11	2.2	119	54.1	14	2.8
96	43.6	11	2.2	120	54.5	14	2.8
97	44.1	11	2.2	121	55.0	14	2.8
98	44.5	11	2.2	122	55.5	14	2.8
99	45	11	2.2	123	55.9	14	2.8
100	45.5	11	2.2	124	56.4	14	2.8
101	45.9	11	2.2	125	56.8	14	2.8
102	46.4	11	2.2	126	57.3	14	2.8

Weight ≥127 lbs/57.7 kg
Tenecteplase -Tissue Plasminogen Activator (Tenecteplase, TNKase) Weight Dose Chart for Stroke Indication Using 50mg Vial (5mg/ml concentration) Dose 0.25 mg/kg Max Dose 25 mg= 5 mL

Patient Weight		Total Dose 0.25mg/Kg to a Max of 25mg	Total Dose IV Push over 5sec	Patient Weight		Total Dose 0.25mg/Kg to a Max of 25 mg	Total Dose IV Push mL over 5sec
lbs	Kg	Dose mg	Dose mg=ml	lbs	Kg	Dose mg	Dose mg=ml
127	57.7	14	2.8	172	78.1	20	4
128	58.2	15	3	173	78.6	20	4
129	58.6	15	3	174	79.0	20	4
130	59.1	15	3	175	79.5	20	4
131	59.5	15	3	176	80.0	20	4
132	60.0	15	3	177	80.4	20	4
133	60.5	15	3	178	80.9	20	4
134	60.9	15	3	179	81.4	20	4
135	61.4	15	3	180	81.8	20	4
136	61.8	15	3	181	82.3	21	4.2
137	62.3	16	3.2	182	82.7	21	4.2
138	62.7	16	3.2	183	83.1	21	4.2
139	63.2	16	3.2	184	83.6	21	4.2
140	63.6	16	3.2	185	84.0	21	4.2
141	64.1	16	3.2	186	84.5	21	4.2

142	64.5	16	3.2
143	65.0	16	3.2
144	65.5	16	3.2
145	65.9	16	3.2
146	66.4	17	3.4
147	66.8	17	3.4
148	67.3	17	3.4
149	67.7	17	3.4
150	68.2	17	3.4
151	68.6	17	3.4
152	69.1	17	3.4
153	69.5	17	3.4
154	70.0	18	3.6
155	70.5	18	3.6
156	70.9	18	3.6
157	71.4	18	3.6
158	71.8	18	3.6
159	72.3	18	3.6
160	72.7	18	3.6
161	73.2	18	3.6
162	73.6	18	3.6
163	74.1	19	3.8
164	74.5	19	3.8
165	75.0	19	3.8
166	75.5	19	3.8
167	75.9	19	3.8
168	76.4	19	3.8
169	76.8	19	3.8
170	77.3	19	3.8
171	77.7	19	3.8
187	85.0	21	4.2
188	85.5	21	4.2
189	85.9	21	4.2
190	86.4	22	4.4
191	86.8	22	4.4
192	87.3	22	4.4
193	87.7	22	4.4
194	88.2	22	4.4
195	88.6	22	4.4
196	89.0	22	4.4
197	89.5	22	4.4
198	90.0	23	4.6
199	90.5	23	4.6
200	91.0	23	4.6
201	91.4	23	4.6
202	92.0	23	4.6
203	92.3	23	4.6
204	93.0	23	4.6
205	93.2	23	4.6
206	93.6	23	4.6
207	94.0	24	4.8
208	94.5	24	4.8
209	95.0	24	4.8
210	95.5	24	4.8
211	95.9	24	4.8
212	96.4	24	4.8
213	96.8	24	4.8
214	97.3	24	4.8
215	97.7	24	4.8
≥216	≥98.2	25 Max	5 Max
